# Progress Toward Poliomyelitis Eradication — Afghanistan, January 2019–July 2020

**DOI:** 10.15585/mmwr.mm6940a3

**Published:** 2020-10-09

**Authors:** Maureen Martinez, Irfan Elahi Akbar, Mufti Zubair Wadood, Hemant Shukla, Jaume Jorba, Derek Ehrhardt

**Affiliations:** ^1^Global Immunization Division, Center for Global Health, CDC; ^2^Polio Eradication Department, World Health Organization, Kabul, Afghanistan; ^3^Polio Eradication Department, World Health Organization, Geneva, Switzerland; ^4^Polio Eradication Department, World Health Organization, Amman, Jordan; ^5^Division of Viral Diseases, National Center for Immunization and Respiratory Diseases, CDC.

Wild poliovirus type 1 (WPV1) transmission is ongoing only in Afghanistan and Pakistan (*1*). Following a decline in case numbers during 2013–2016, the number of cases in Afghanistan has increased each year during 2017–2020. This report describes polio eradication activities and progress toward polio eradication in Afghanistan during January 2019–July 2020 and updates previous reports (*2*,*3*). Since April 2018, insurgent groups have imposed bans on house-to-house vaccination. In September 2019, vaccination campaigns in areas under insurgency control were restarted only at health facilities. In addition, during March–June 2020, all campaigns were paused because of the coronavirus disease 2019 (COVID-19) pandemic. The number of WPV1 cases reported in Afghanistan increased from 21 in 2018 to 29 in 2019. During January–July 2020, 41 WPV1 cases were reported as of August 29, 2020 (compared with 15 during January–July 2019); in addition, 69 cases of circulating vaccine-derived poliovirus type 2 (cVDPV2), and one case of ambiguous vaccine-derived poliovirus type 2 (aVDPV2) (isolates with no evidence of person-to-person transmission or from persons with no known immunodeficiency) were detected. Dialogue with insurgency leaders through nongovernmental and international organizations is ongoing in an effort to recommence house-to-house campaigns, which are essential to stopping WPV1 transmission in Afghanistan. To increase community demand for polio vaccination, additional community health needs should be addressed, and polio vaccination should be integrated with humanitarian services.

## Immunization Activities

In September 2015, wild poliovirus type 2 was declared to be globally eradicated, and a single dose of injectable inactivated poliovirus vaccine (IPV, containing inactivated vaccine virus types 1, 2, and 3) was introduced into the routine immunization program in Afghanistan. In April 2016, type 2 oral poliovirus vaccine (OPV) was withdrawn through a globally synchronized switch from trivalent OPV (tOPV, containing Sabin-strain types 1, 2, and 3) to bivalent OPV (bOPV, containing types 1 and 3.) The World Health Organization (WHO) and United Nations Children’s Fund (UNICEF) estimated national routine vaccination coverage of children aged <12 months with the third dose of bOPV in Afghanistan was 73% in 2018 and 2019. Estimated 1-dose IPV coverage in 2019 was 66% (*4*). In 2019, 69% of children aged 6–23 months with nonpolio acute flaccid paralysis (NPAFP) nationwide had a history of receipt of 3 OPV doses (OPV3) through routine immunization services, a proxy indicator of OPV3 coverage. The proportion of children with NPAFP who never received OPV through routine or supplementary immunization activities (SIAs)* was 1% nationally in 2019, with higher percentages in the southern provinces of Kandahar (9%) and Uruzgan (25%), and the eastern province of Kunar (7%).

During January 2019–July 2020, where vaccination campaigns were allowed, 5 national immunization days (NIDs), 5 subnational immunization days (SNID), two WPV1 case response campaigns, and one cVDPV2 case response campaign SIAs targeted children aged <5 years for receipt of monovalent OPV type 1 (mOPV1, containing Sabin-strain type 1), bOPV, or monovalent OPV type 2 (mOPV2, containing Sabin-strain type 2), including NIDs targeted 9,999,227 children aged <5 years. During SIAs, IPV was administered to 554,211 (87%) children targeted in the districts at highest risk for poliovirus transmission.

To reach every child with OPV, the polio program conducts house-to-house SIAs whenever feasible. Children who are missed during campaigns are classified as inaccessible if they live in areas with security challenges to access or where campaigns are banned. Children are considered missed but accessible when they are not vaccinated because of campaign quality issues. SIAs were banned in all areas controlled by the insurgency in April 2018. Since September 2019, vaccination in these areas has only been permitted at health facilities or insurgency-approved fixed posts.

According to reported administrative data, 449,756 (4%) children aged <5 years in Afghanistan were inaccessible during the March 2019 NID. The number increased to 2,844,197 (28%) during the November 2019 NID and was 2,655,821 (27%) during the January 2020 NID. After a 5-month COVID-19–related pause in SIAs, a cVDPV2 response campaign with mOPV2 was conducted in July 2020 in the eastern region, where 107,768 (10%) of 1,101,740 targeted children were inaccessible. During these SIAs, the numbers of children reported as accessible but missed resulting from campaign quality failures were 399,969 (4%) in March 2019, 299,977 (2%) in November 2019 and January 2020, and 22,035 (2%) in July 2020.

Lot quality assurance sampling (LQAS)^†^ surveys assess SIA quality in areas where permitted, with more accuracy than convenience-sampled, post-SIA monitoring surveys. Depending on the number of unvaccinated children among 60 children surveyed, SIAs in districts are marked either “passed” at a 90% threshold or “failed.” The proportion of districts with failed LQAS SIAs was 29% in April 2019, 15% in November 2019, 24% in January 2020, and 40% in July 2020.

Children aged ≤10 years are also targeted for vaccination along major travel routes throughout Afghanistan, at transit points from inaccessible areas, and at border-crossing points with Iran and Pakistan. During January 2019–July 2020, 24,009,626 doses of bOPV were administered at transit points and 1,296,109 doses at border crossings. Starting in March 2019, documented annual bOPV vaccination was required for all persons entering Afghanistan from Pakistan, resulting in an additional 551,837 doses administered to persons aged >10 years.

## Poliovirus Surveillance

**Acute flaccid paralysis surveillance**. Detection of two or more NPAFP cases per 100,000 persons aged <15 years indicates surveillance sufficiently sensitive to detect a poliovirus case; to assess the ability to detect poliovirus among those with acute flaccid paralysis (AFP), 80% of AFP cases should have adequate stool specimens collected.^§^ The Afghanistan AFP surveillance network includes 2,501 health facilities and 38,140 community volunteers. In 2019, the national NPAFP rate was 18 per 100,000 persons aged <15 years with regional rates ranging from 12 to 26 (Table). The percentage of AFP cases with adequate specimens was 94% (regional range = 90%–98%).

**TABLE:** Acute flaccid paralysis (AFP) surveillance performance indicators and reported cases of wild poliovirus (WPV1) and vaccine-derived poliovirus type 2 (VDPV2),* by region and period — Afghanistan, January 2019–July 2020†

Region	AFP surveillance indicators (2019)			No. of WPV1 cases reported			No. of cVDPV2 cases reported			No. of aVDPV2 cases reported		
	No. of AFP cases	NPAFP rate^§^	% AFP cases with adequate stool specimens^¶^	2019		2020	2019		2020	2019		2020
				Jan–Jul	Aug–Dec	Jan–Jul	Jan–Jul	Aug–Dec	Jan–Jul	Jan–Jul	Aug–Dec	Jan–Jul
**All regions**	**3,768**	**18**	**94**	**15**	**14**	**41**	**0**	**0**	**69**	**0**	**0**	**1**
Badakhshan	74	12	91	0	0	1	0	0	1	0	0	0
Central	646	14	98	0	0	0	0	0	2	0	0	0
Eastern	552	26	95	1	1	2	0	0	62	0	0	1
Northeastern	497	21	94	0	1	0	0	0	0	0	0	0
Northern	387	15	92	0	0	1	0	0	0	0	0	0
Southeastern	372	18	97	0	2	0	0	0	2	0	0	0
Southern	632	17	90	14	6	30	0	0	1	0	0	0
Western	608	21	94	0	4	7	0	0	1	0	0	0

**Abbreviations:** aVDPV2 = ambiguous vaccine-derived poliovirus type 2; cVDPV2 = circulating vaccine-derived poliovirus type 2; NPAFP = nonpolio acute flaccid paralysis.

* aVDPVs are isolates with no evidence of person-to person transmission or from persons with no known immunodeficiency; cVDPVs are isolates for which there is evidence of person-to-person transmission in the community.

^†^ Data current as of August 29, 2020.

^§^ Cases per 100,000 persons aged <15 years. The target for the nonpolio AFP rate indicator is ≥2 NPAFP cases per 100,000 persons aged <15 years.

^¶^ Surveillance target is that ≥80% of AFP cases have adequate stool specimens collected. Adequate stool specimens are defined as two stool specimens of sufficient quality for laboratory analysis, collected ≥24 hours apart, both within 14 days of paralysis onset, and arriving in good condition at a World Health Organization–accredited laboratory with reverse cold chain maintained, without leakage or desiccation, and with proper documentation.

**Environmental surveillance.** Supplementary poliovirus surveillance in Afghanistan is conducted through systematic sampling of sewage at 23 sites in 11 provinces and virologic testing. WPV1 was detected in 83 (25%) of 336 environmental surveillance (ES) specimens in 2018, 56 (23%) of 259 in 2019, and 26 (10%) of 249 in 2020 (as of August 29). All WPV1 ES detections in 2019 were in Helmand, Kandahar (southern region) and Nangarhar (eastern) provinces. To date in 2020, WPV1 ES detections were in these provinces plus Khost (southeastern) and Herat (western). In 2020, 56 of 249 (23%) ES specimens tested positive for cVDPV2 in Kandahar and Helmand (southern), Nangarhar and Kunar (eastern), Khost (southeastern), and Kabul (central) provinces.

## Epidemiology of Poliovirus Cases

**FIGURE 1:**
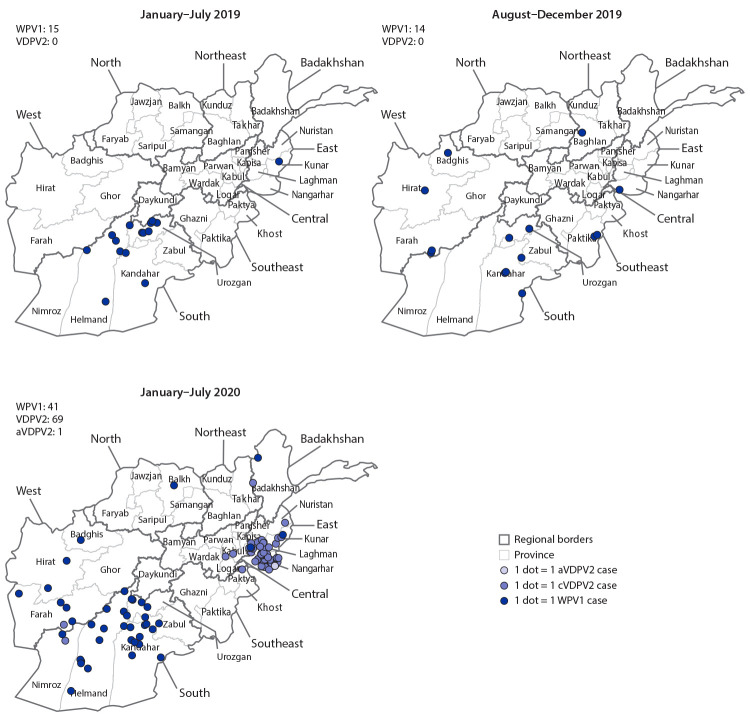
Cases of wild poliovirus type 1 (WPV1) and vaccine- derived poliovirus type 2 (VDPV2),* by providence — Afghanistan, January 2019–July 2020<Fig_Large></Fig_Large> **Abbreviations:** aVDPV2 = ambiguous vaccine-derived poliovirus type 2; cVDPV2 = circulating vaccine-derived poliovirus type 2. * aVDPVs are isolates with no evidence of person-to person transmission or from persons with no known immunodeficiency; cVDPVs are isolates for which there is evidence of person-to-person transmission in the community.

**FIGURE 2:**
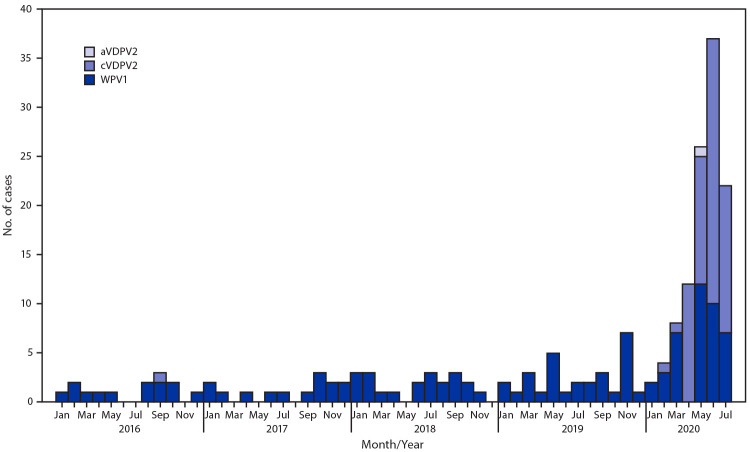
Number of wild poliovirus type 1 (WPV1) cases (n = 91) and vaccine- derived poliovirus type (V2PV2)* cases (n = 71) — Afghanistan, January 2016–July 2020^†^ <Fig_Large></Fig_Large> **Abbreviations:** aVDPV2 = ambiguous vaccine-derived poliovirus type 2; cVDPV2 = circulating vaccine-derived poliovirus type 2. * aVDPVs are isolates with no evidence of person-to person transmission or from persons with no known immunodeficiency; cVDPVs are isolates for which there is evidence of person-to-person transmission in the community. ^†^ Data as of August 29, 2020.

During 2019, 29 WPV1 cases were reported from 20 districts in 10 provinces, compared with 21 WPV1 cases reported from 14 districts in six provinces in 2018. During January–July 2020, 41 WPV1 cases were reported from 30 districts in 12 provinces compared with 15 from 11 districts in four provinces during the same period in 2019 (Table) (Figure 1) (Figure 2). Among the 70 WPV1 cases reported during January 2019–July 2020, 54 (77%) were in children aged <36 months. Nineteen (27%) of the 70 patients had never received OPV through routine immunization or SIAs, 15 (21%) had received 1 or 2 doses, and 36 (51%) had received ≥3 doses each; 46 (66%) had never received OPV through routine immunization but had received ≥1 SIA doses.

Genomic sequence analysis of the region encoding capsid protein VP1 of poliovirus isolates identified evidence of multiple episodes of cross-border transmission between Afghanistan and Pakistan during 2018–2020, with sustained local transmission in both countries. During January 2019–July 2020, 13 (20%) of 66 WPV1 isolates from AFP patients and 17 (22%) of 77 WPV1 ES isolates in Afghanistan had closest genetic links to earlier WPV1 isolates from Pakistan; the remaining were most closely linked to patient and ES isolates within Afghanistan. During January 2018–July 2020, four WPV1 genetic clusters (≥95% sequence identity) were detected among AFP cases. Although transmission in eastern and southern provinces is largely from distinct genetic clusters, three WPV1 cases were identified in the south from clusters originally identified in the east. During January 2019–July 2020, 13 orphan WPV1 viruses isolated from ES or AFP cases (those with ≥1.5% divergence from their closest genetic match, i.e., ≤98.5% of a match) were detected, signaling gaps in AFP surveillance.

During January–July 2020, 69 cVDPV2 cases and one aVDPV2 case were reported from 34 districts in 10 provinces, 57 (81%) of which occurred in children aged <36 months, and 68 (97%) of which are genetically related to the PAK-GB-1 emergence first detected in Gilgit-Baltistan, Pakistan. The remaining two cases were classified as a new Afghanistan cVDPV2 emergence (AFG-NGR-1) first detected in Nangarhar province and an aVDPV2 with no genetic linkage to known polioviruses.

## Discussion

On August 25, 2020, the WHO African Region was certified WPV-free, the fifth of six WHO regions to be certified, leaving only the Eastern Mediterranean Region with endemic WPV1 circulation in Afghanistan and Pakistan. Afghanistan has interrupted internal transmission of WPV1 for short periods in the past (*5*). Widespread bans on house-to-house vaccination in insurgency-held areas since April 2018 have resulted in increasing numbers of WPV1 cases. In 2020, COVID-19 pandemic mitigation efforts in Afghanistan halted SIAs for 5 months, compounding the existing access and SIA quality issues and resulting in increased numbers of susceptible children.

The cVDPV2 Pakistan outbreak that began in 2019 rapidly spread to Afghanistan and is growing. The polio program was able to resume mOPV2 outbreak response campaigns in July 2020; however, the lack of full access for house-to-house immunization will limit the effectiveness of these campaigns.

The primary barrier to interrupting poliovirus transmission in Afghanistan is the number of inaccessible children in insurgency-held areas. Dialogue with insurgency leaders through nongovernmental and international organizations to regain house-to-house access, which was successful in earlier years, needs enhanced efforts and new partners. In the interim, focus must be placed on finding and strengthening alternatives to SIAs for vaccinating children against polio. Before being aborted at the start of the COVID-19 pandemic, the country was in the process of rolling out integrated services to address widespread health demands beyond vaccination in polio-priority areas and to integrate OPV use into other health programs; provision of broad services should be fully implemented. With SIA resumption, partnering with other health sectors to offer multiantigen vaccination alongside other health services of high priority will increase community polio vaccination demand and coverage.

**Summary**

**What is already known about this topic?**

Wild poliovirus circulation continues in Afghanistan.

**What is added by this report?**

After approximately 2 years of campaign bans by the insurgency coupled with the COVID-19 pandemic, wild poliovirus circulation has increased during 2019–2020, and a new vaccine-derived poliovirus type 2 outbreak began in 2020.

**What are the implications for public health practice?**

Polio vaccination must be incorporated more broadly into public health services in order to reach every child. New partners should be engaged in discussions with local leaders to facilitate the recommencement of nationwide house-to-house campaigns.

## Footnotes

* SIAs are mass house-to-house campaigns targeting children aged <5 years with OPV, regardless of their vaccination history.

† Lot quality assurance sampling is a rapid method used to assess the quality of vaccination activities after SIAs in predefined areas such as health districts (referred to as “lots”), using a small sample size. Lot quality assurance sampling involves dividing the population into lots and ascertaining receipt of vaccination by randomly selected persons within each lot. If the number of unvaccinated persons in the sample exceeds a predetermined value, then the lot is classified as having an unsatisfactory level of vaccination coverage, and mop-up activities are recommended. If the threshold of ≥90% is met (Afghanistan program guidelines have recently increased the threshold from ≥80%), the area or district is classified as having passed, although mop-up activities might still be indicated in certain areas.

§ Surveillance target is ≥80% of AFP cases with adequate stool specimens collected. Adequate stool specimens are defined as two stool specimens of sufficient quality for laboratory analysis, collected ≥24 hours apart, both within 14 days of paralysis onset, and arriving in good condition at a World Health Organization–accredited laboratory with reverse cold chain maintained, without leakage or desiccation, and with proper documentation.
